# Supplementary material for: ROS-Mediated Nematocidal Activity and Reproductive Toxicity of Herbal Extracts in Caenorhabditis elegans
Source: Nutrients. 2025 Oct 23;17(21):3337. doi: 10.3390/nu17213337 (PMC12608140; doi:10.3390/nu17213337)
Supplement: Supplementary file 1 [file nutrients-17-03337-s001.zip › nutrients-3903433-supplementary/Supplemtary Figure 1,2,3,4.pdf]

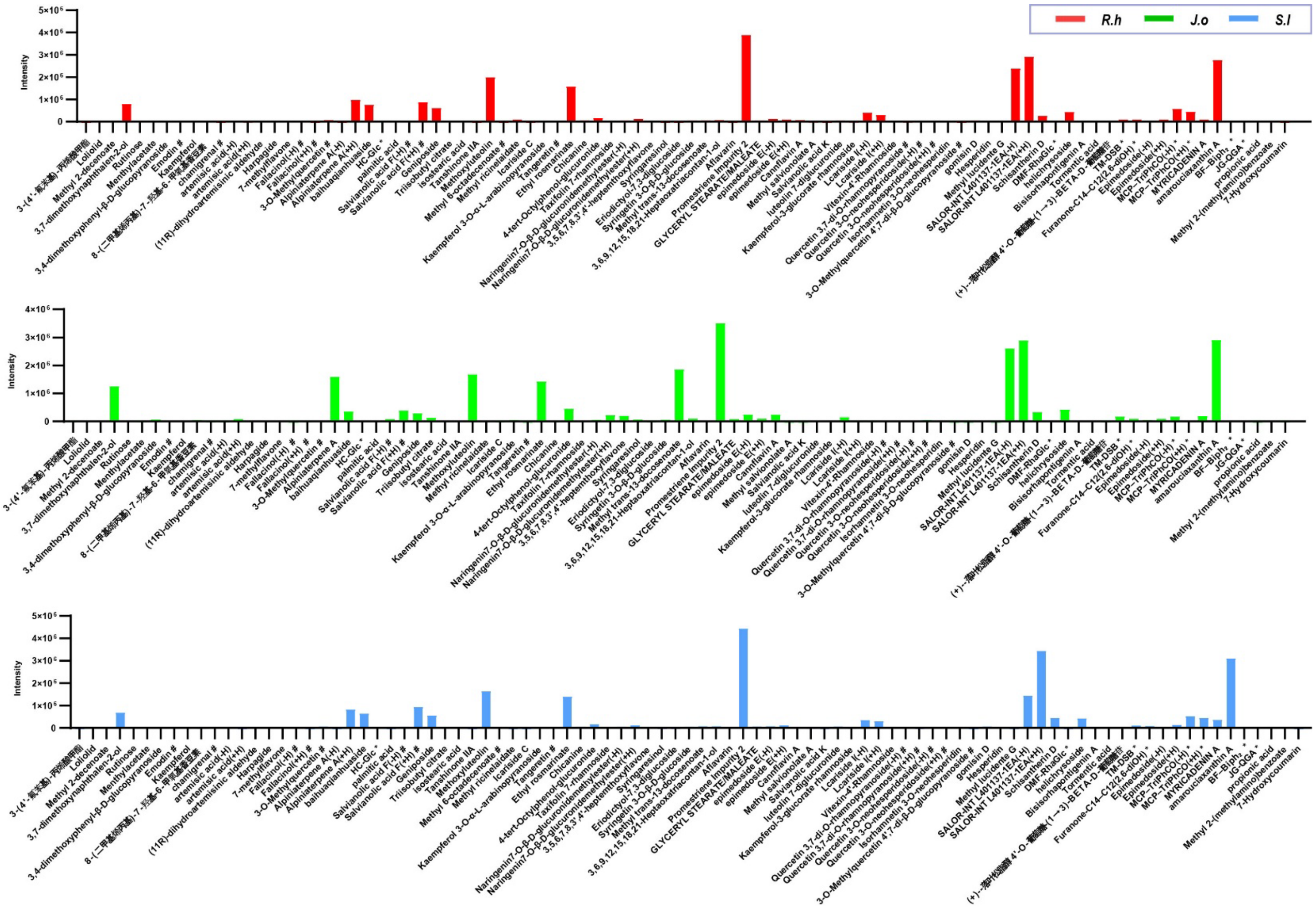

Supplementary Figure S1. LC–MS profiling of 79 compounds in *Ruscus hyrcanus* extract (RHE), *Juniperus oblonga* extract (JOE), and *Stachys lavandulifolia* extract (SLE). Peak intensities (y-axis) are shown for 78-79 compounds detected across all three herb extracts (x-axis). RHE, JOE and SLE are represented by red, green, and blue traces, respectively. # indicates isomers, and * denotes a shortened form of the full compound name. The 93 complete list of compounds is provided in Supplementary Table S1-S3. List of 78 shared compounds can be found in Supplementary Table S4. Full name and isomers can be found in Supplementary Table S5

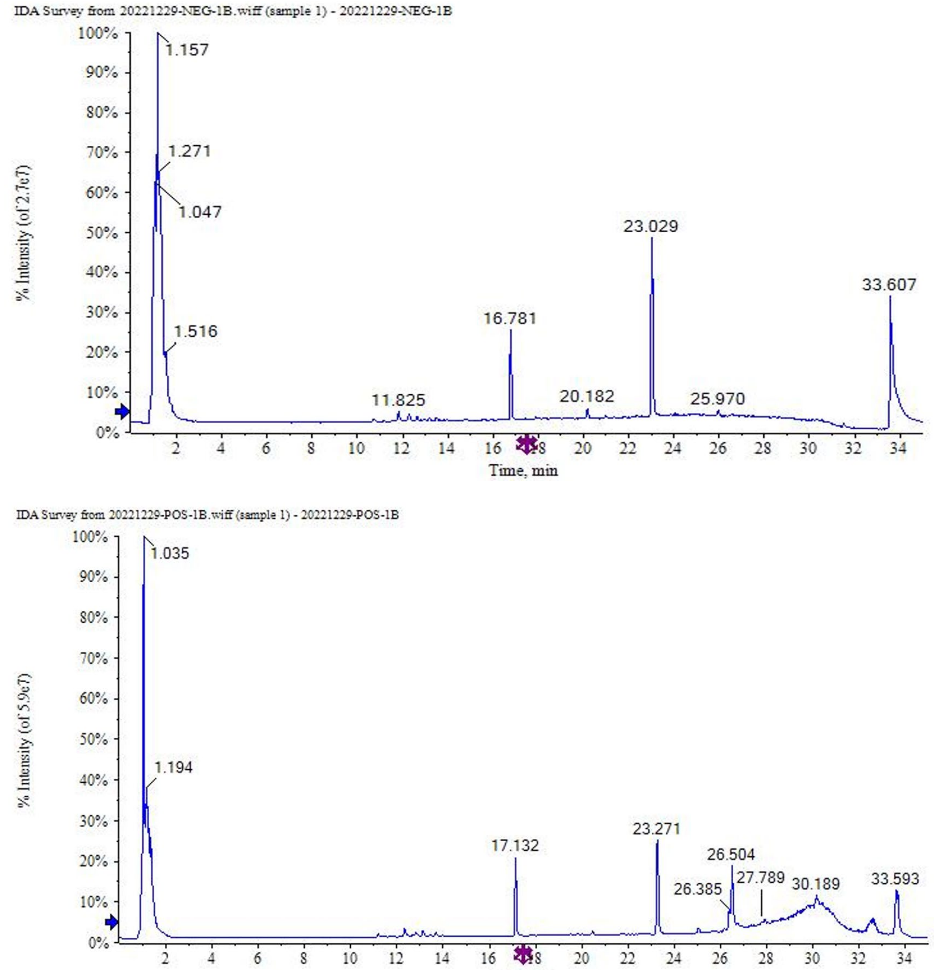

Supplementary Figure S2. Chromatograms of *R.h* obtained in positive (+H) and negative (–H) ion modes. The y-axis represents ion intensity, and the x-axis indicates retention time (min). Most compounds were eluted within approximately 0–35 minutes under the current chromatographic conditions.

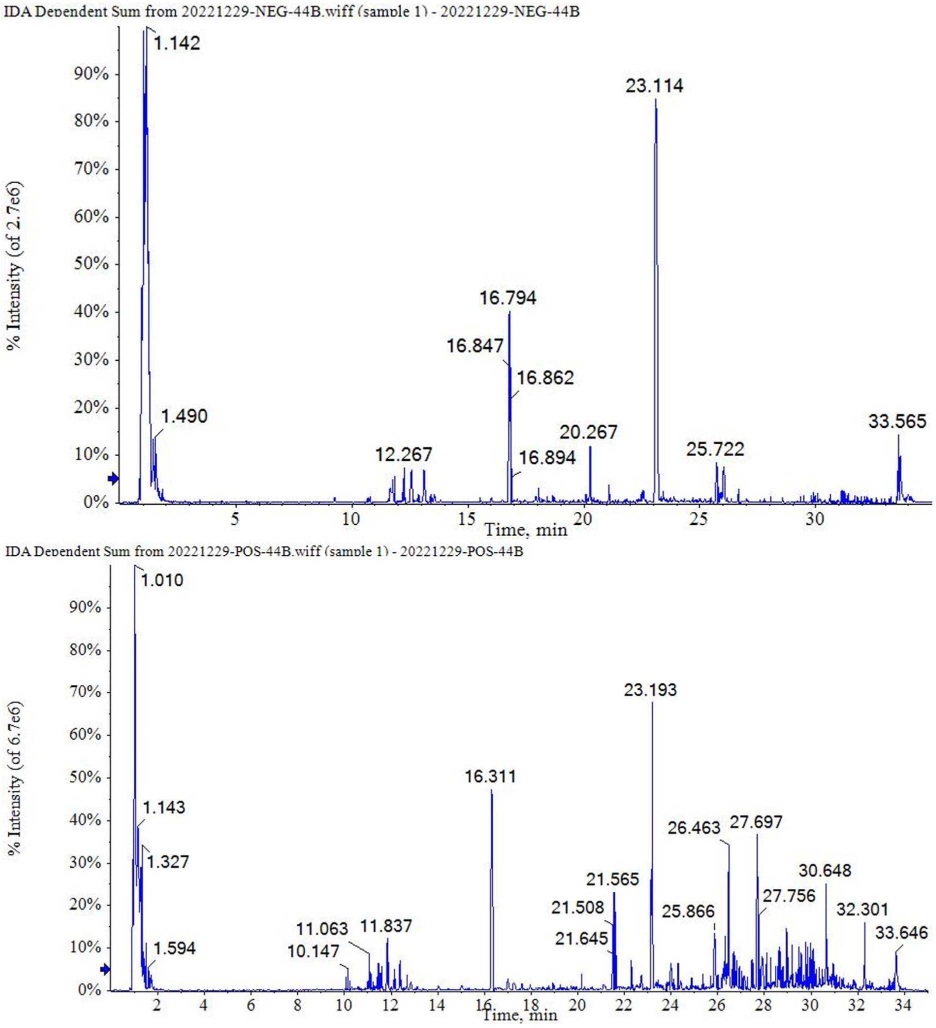

Supplementary Figure S3. Chromatograms of *J.o* obtained in positive (+H) and negative (–H) ion modes. The y-axis represents ion intensity, and the x-axis indicates retention time (min). Most compounds were eluted within approximately 0–35 minutes under the current chromatographic conditions.

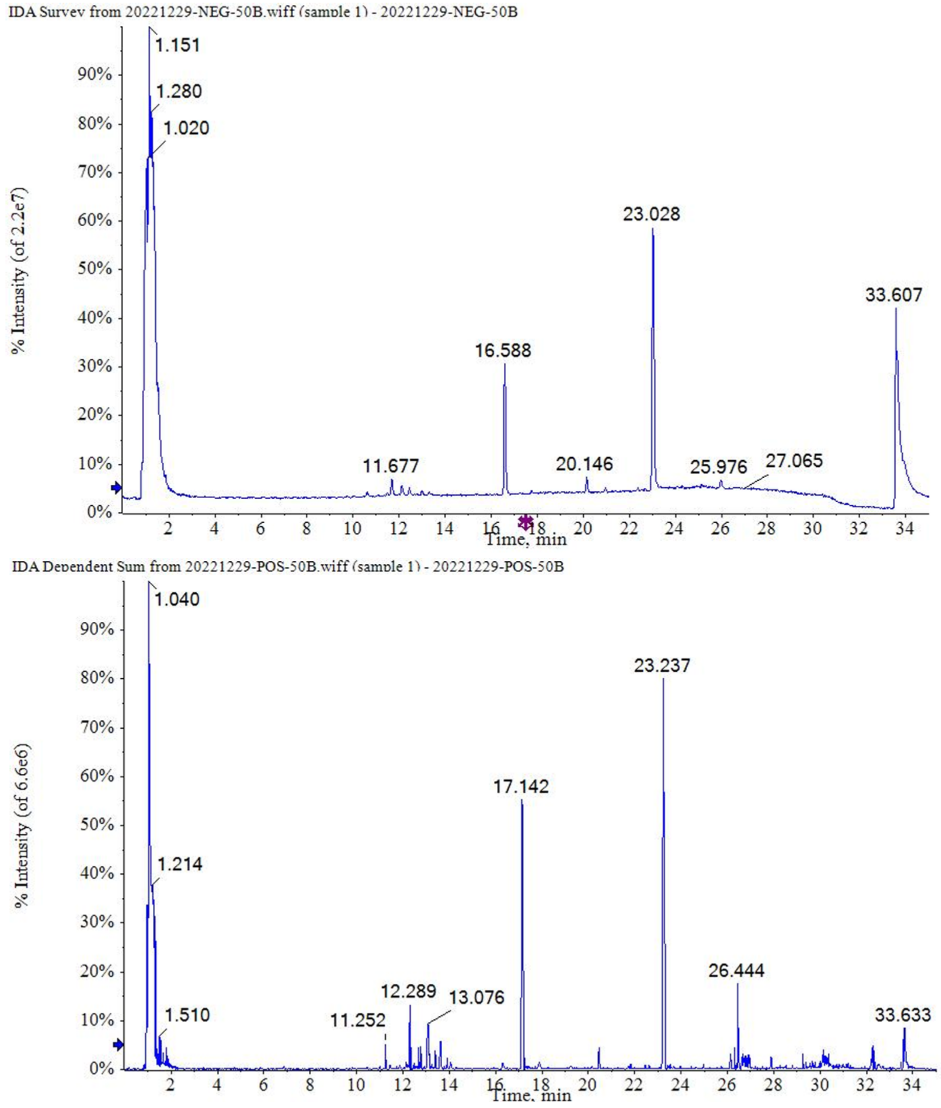

Supplementary Figure S4. Chromatograms of *S.l.* obtained in positive (+H) and negative (–H) ion modes. The y-axis represents ion intensity, and the x-axis indicates retention time (min). Most compounds were eluted within approximately 0–35 minutes under the current chromatographic conditions.
